# A Subnano-g Electrostatic Force-Rebalanced Flexure Accelerometer for Gravity Gradient Instruments

**DOI:** 10.3390/s17112669

**Published:** 2017-11-18

**Authors:** Shitao Yan, Yafei Xie, Mengqi Zhang, Zhongguang Deng, Liangcheng Tu

**Affiliations:** 1 MOE Key Laboratory of Fundamental Physical Quantities Measurement & Hubei Key Laboratory of Gravitation and Quantum Physics, School of Physics, Huazhong University of Science and Technology, Wuhan 430074, China; yanshitao@hust.edu.cn (S.Y.); xieyaphe@hust.edu.cn (Y.X.); mqzhang@hust.edu.cn (M.Z.); dzg_109@hust.edu.cn (Z.D.); 2 Institute of Geophysics, Huazhong University of Science and Technology, Wuhan 430074, China

**Keywords:** flexure accelerometer, subnano-g resolution, capacitive displacement transducer, electrostatic force-rebalanced, rotating accelerometer gravity gradient instrument

## Abstract

A subnano-g electrostatic force-rebalanced flexure accelerometer is designed for the rotating accelerometer gravity gradient instrument. This accelerometer has a large proof mass, which is supported inversely by two pairs of parallel leaf springs and is centered between two fixed capacitor plates. This novel design enables the proof mass to move exactly along the sensitive direction and exhibits a high rejection ratio at its cross-axis directions. Benefiting from large proof mass, high vacuum packaging, and air-tight sealing, the thermal Brownian noise of the accelerometer is lowered down to less than 0.2 $\mathrm{ng}/\sqrt{\mathrm{Hz}}$ with a quality factor of 15 and a natural resonant frequency of about 7.4 Hz. The accelerometer’s designed measurement range is about ±1 mg. Based on the correlation analysis between a commercial triaxial seismometer and our accelerometer, the demonstrated self-noise of our accelerometers is reduced to lower than 0.3 $\mathrm{ng}/\sqrt{\mathrm{Hz}}$ over the frequency ranging from 0.2 to 2 Hz, which meets the requirement of the rotating accelerometer gravity gradiometer.

## 1. Introduction

Novel gravity gradiometer is of great importance in geodetic surveys, resource explorations, and inertial navigations [1,2,3,4,5]. Over the last century, different technologies have been applied to the developments of gravity gradiometers. In general, these technologies can be divided into five categories, including torsion balance-based [6], rotating accelerometer-based [7,8], superconducting [9], cold atomic interferometer-based [10], and MEMS gravity gradiometers [11,12]. Rotating accelerometer gravity gradient instrument (GGI) [7,8] was firstly developed by Bell Aerospace in the 1970s. To date, it is the only commercially available gravity gradiometer that has accomplished flight test in terrestrial space with the desired sensitivity. The most critical component of a GGI is the accelerometer that features a single sensitive axis, extra-low noise over the rotating frequency range, high rejection ratio at the cross-axis directions, low-temperature coefficient of the scale factor, and suitable volume for its carrier (e.g., a turntable). For a typical example, the accelerometer that is employed in the Bell’s GGI was upgraded from Bell model VII electromagnetic accelerometer, having an improved resolution from 0.6 $\mathrm{ng}/\sqrt{\mathrm{Hz}}$ at 0.0167 Hz to 0.01 $\mathrm{ng}/\sqrt{\mathrm{Hz}}$ at 1 Hz on the null bias mode [7]. 

In order to implement a horizontal GGI of 10 $E/\sqrt{\mathrm{Hz}}$ ($1\;E=10^{-9}/s^{2}\approx 10^{-10}\;g/m$) resolution with a baseline of 0.3 m, an accelerometer with a self-noise lower than 0.3 $\mathrm{ng}/\sqrt{\mathrm{Hz}}$ at the rotating frequency range of 0.2 to 2 Hz and a measurement range of at least ±1 mg shall be developed. Most reported accelerometers with the self-noise of lower than 0.3 $\mathrm{ng}/\sqrt{\mathrm{Hz}}$ are manufactured by conventional high-precision machining to achieve the large proof mass. This usually results in a low noise equivalent acceleration (NEA) of the mechanical probe. For example, the STAR electrostatic accelerometer [13], designed for the CHAllenging Minisatellite Payload (CHAMP) mission, indicated a measurement range of ±1 mg and a resolution of $<1\;\mathrm{ng}/\sqrt{\mathrm{Hz}}$ over the frequency range of 0.1 mHz to 0.1 Hz. The SuperSTAR electrostatic accelerometer [13], derived from STAR, was developed for the Gravity Recovery And Climate Experiment (GRACE) mission and had an improved resolution of 0.01 $\mathrm{ng}/\sqrt{\mathrm{Hz}}$ over the frequency range of 0.1 mHz to 0.1 Hz and a measurement range of ±5 μg. In addition, designed for the electrostatic gravity gradiometer, the Gravity field and steady-state Ocean Circulation Explorer (GOCE) accelerometers that were based on electrostatic control technology demonstrated a resolution of 0.2 $\mathrm{pg}/\sqrt{\mathrm{Hz}}$ over the frequency range of 5 mHz to 0.1 Hz, with only ±0.65 μg measurement range [13]. Unfortunately, these electrostatic accelerometers are specially designed to work in outer space and offer a small measurement range. Other commercial seismometers, such as Nanometrics’ Trillium compact HP, Guralp’s CMG-3T, and Streckeisen’s STS-2 have bandwidths of 8 mHz to 100 Hz, 8 mHz to 50 Hz and 8 mHz to >50 Hz, and self-noises of 0.3 $\mathrm{ng}/\sqrt{\mathrm{Hz}}$, 0.03 $\mathrm{ng}/\sqrt{\mathrm{Hz}}$, and 0.01 $\mathrm{ng}/\sqrt{\mathrm{Hz}}$ [14,15,16,17], respectively. But, they are hardly used in GGI due to their large volumes and limited measurement ranges. More importantly, the electromagnetic actuators consist of high-temperature-coefficient permanent magnets for operation, making their scale factor more sensitive to temperatures than the electrostatic accelerometers.

In this paper, we demonstrate a single-axis force-rebalanced flexure accelerometer that can have practical applications in GGIs. This accelerometer is designed based on high precision capacitive sensing and electrostatic control technology. Self-noise of the accelerometer is measured to be less than 0.3 $\mathrm{ng}/\sqrt{\mathrm{Hz}}$ in the frequency range from 0.2 to 2 Hz. The key improvement of this design is to use two pairs of symmetrical parallel leaf springs to inversely support the large proof mass, and a very high rejection ratio is successfully achieved at the cross-axis direction. The accelerometer meets the requirement for GGIs and we foresee that this accelerometer will be useful to the development of GGIs and other high-end sensors in the near future.

## 2. Structure Design and Noise Analysis of the Electrostatic Accelerometer

### 2.1. Working Principle of the Electrostatic Accelerometer

**Figure 1:**
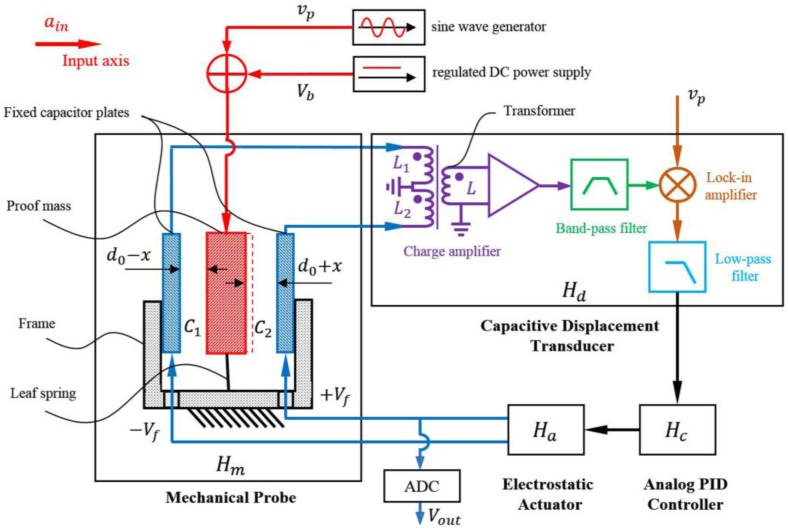
Schematic of the electrostatic accelerometer system. $a_{in}$ is the external input acceleration. $d_{0}$ is the gap of the balanced capacitor $C_{0}$ and x is the displacement of the proof mass. $C_{1}$ and $C_{2}$ are the differential detecting capacitors. $L_{1}$ and $L_{2}$ are the inductances of the transformer’s primary windings, while L is that of the secondary. $H_{m}$, $H_{d}$, $H_{c}$, and $H_{a}$ denote the transfer functions of the mechanical probe, the capacitive displacement transducer, the analog proportional-integral-differential (PID) controller and the electrostatic actuator, respectively.

A block diagram of the electrostatic accelerometer system is shown in Figure 1. The electrostatic accelerometer consists of a mechanical probe, a high precision capacitive displacement transducer, an analog proportional-integral-differential (PID) controller, and a high-performance electrostatic actuator. Here, $H_{m}$, $H_{d}$, $H_{c}$, $H_{a}$ represent the transfer functions of these four stages, respectively. The mechanical probe of this electrostatic accelerometer is derived from a typical spring-mass structure. The rectangular proof mass is inversely supported by a set of leaf springs. Two fixed capacitor plates, together with the proof mass, form two gap-varying differential capacitors, which can transfer the external acceleration into a differential output of the capacitors, namely $C_{1}$ and $C_{2}$. The capacitive displacement transducer consists of four parts: a charge amplifier, a band-pass filter, a lock-in amplifier, and a low-pass filter [18,19]. The driving voltage applied to the proof mass is composed of an AC pumping voltage $v_{p}$ and a DC bias voltage $V_{b}$. The analog PID controller and the electrostatic actuator together constitute an electrostatic closed-loop feedback. Taking the output of the low-pass filter as the input of the PID controller, an appropriate voltage is produced by the PID controller and is then sent to the electrostatic actuator for further amplification. Finally, two opposite feedback voltages $\pm V_{f}$ are obtained and are then applied to the two fixed capacitor plates to generate two opposite electrostatic forces. The applied $V_{f}$ is sampled by a 24-bits Analog to Digital Converter (ADC), which is the output voltage $V_{out}$ of the accelerometer.

Once an external acceleration is applied to the input axis direction, there would be a displacement of the proof mass with respect to the frame that is caused by the input acceleration. Because the proof mass is sandwiched between the two capacitor plates that are fixed to the frame, this displacement will introduce variations of the capacitance of the two differential capacitors $C_{1}$ and $C_{2}$. This variation is then sensed by the capacitive displacement transducer. After that, the PID controller obtains the displacement information and leads the electrostatic actuator to generate two opposite electrostatic forces, which eventually drive the proof mass back to its null position. 

Since the spring-mass structure has a typical spring-mass-damper system, according to Newton’s second law of motion, the equation of motion of the electrostatic force-rebalanced flexure accelerometer can be expressed as [18,19,20,21]

(1) $$m\ddot{x}+\gamma \dot{x}+k_{0}x=ma_{in}+F_{e},$$

where m is the mass of the proof mass, γ is the damping coefficient, $k_{0}$ is the stiffness coefficient of the spring, $a_{in}$ is the input acceleration, and $F_{e}$ is the electrostatic feedback force. In the closed-loop mode, the electrostatic force $F_{e}$ is equivalent to the input force $ma_{in}$ acted on the proof mass to keep the proof mass still and always remain at its null position. Therefore, the value of the electrostatic feedback force will be taken as a measure of the input acceleration, and is noted as the output acceleration $a_{out}$ of the accelerometer. According to the block diagram, as shown in Figure 1, the relationship between $a_{out}$ and $a_{in}$ can be written as

(2) $$a_{out}=a_{in}\frac{H_{m}H_{d}H_{c}H_{a}}{1+H_{m}H_{d}H_{c}H_{a}}$$

Because the gain $H_{m}H_{d}H_{c}H_{a}$ is much bigger than 1 within the working bandwidth of the accelerometer, the output acceleration $a_{out}\approx a_{in}$. Therefore, the input acceleration $a_{in}$ can be easily obtained by collecting the feedback voltage $V_{f}$, which is denoted as the output voltage $V_{out}$ of the accelerometer:

(3) $$a_{in}\approx V_{out}H_{a},$$

where $H_{a}$ is the voltage-to-acceleration transfer function of the electrostatic actuator.

As shown in Figure 1, a DC bias voltage $V_{b}$ and an AC pumping voltage $v_{p}$ are applied to the proof mass, for linearization of the electrostatic feedback actuator and capacitance sensing, respectively. Furthermore, two opposite feedback voltages $\pm V_{f}$ are applied to the two fixed capacitor plates to generate a difference of potential between the proof mass and the fixed plates, in order to restore the proof mass to its null position. Those voltages would contribute to an electrostatic field between the proof mass and the fixed plates, and yield an electrostatic feedback force on the proof mass [21,22,23,24]:

(4) $$F_{e}=-\frac{2C_{0}}{d_{0}}V_{b}V_{f}+\frac{2C_{0}}{d_{0}{}^{2}}\left(v_{p-rms}{}^{2}+V_{b}{}^{2}+V_{f}{}^{2}\right)x,$$

where $v_{p-rms}$ is the root mean square (RMS) value of the pumping voltage $v_{p}$. The first item in Equation (4) is the effective electrostatic feedback force, and hence the output acceleration can be written as

(5) $$a_{out}\approx \frac{2C_{0}}{md_{0}}V_{b}V_{f},$$

Thus, the voltage-to-acceleration transfer function $H_{a}$ can be written as

(6) $$H_{a}=\frac{2C_{0}}{md_{0}}V_{b}.$$

The reciprocal of $H_{a}$ is the so-called scale factor K of the accelerometer, namely $K=1/H_{a}$.

The second item in Equation (4) is the so-called back-action force, which pushes the proof mass away from its null position and hence could be considered as an equivalent electrostatic spring with a negative stiffness as [18,21]

(7) $$k_{e}=\frac{2C_{0}}{d_{0}{}^{2}}\left(v_{p-rms}{}^{2}+V_{b}{}^{2}+V_{f}{}^{2}\right).$$

The spring stiffness of the spring-mass structure in the closed-loop mode is hence reduced to

(8) $$k^{\prime }=k_{0}-k_{e}.$$

Equation (8) implies that the effective stiffness of the spring-mass system can be adjusted by changing the electrostatic negative stiffness in the closed-loop mode, in which the stiffer springs are used to achieve the same effect as with softer springs in the open-loop mode. 

In order to meet the requirements of GGIs on the accelerometer, it is of the utmost importance to achieve ultra-low noise. The overall NEA of an accelerometer can be generally divided into two parts

(9) $$\delta a_{total-n}=\sqrt{\left(\delta a_{m-n}{}^{2}+\delta a_{e-n}{}^{2}\right)},$$

in which $\delta a_{m-n}$ denotes the mechanical NEA of the spring-mass structure and $\delta a_{e-n}$ is the electronic NEA of the sensing and feedback electronic circuit. As an accelerometer with the self-noise lower than 0.3 $\mathrm{ng}/\sqrt{\mathrm{Hz}}$ is required, the $\delta a_{m-n}$ should be below 0.3 $\mathrm{ng}/\sqrt{\mathrm{Hz}}$. 

### 2.2. Design and Model Analysis of the Spring-Mass Structure

For a typical spring-mass-damper system, the mechanical NEA of the spring-mass structure in a unit bandwidth at all of the frequencies can be written as [21]

(10) $$\delta a_{m-n}=\sqrt{\frac{4k_{B}T\omega_{0}}{mQ}},$$

where $k_{B}$ is the Boltzmann constant, T is the temperature in Kelvin unit, $\omega_{0}=\sqrt{k_{0}/m}$ is the resonant angular frequency, and $Q=\frac{m\omega_{0}}{\gamma }$ is the quality factor of the spring-mass structure.

According to Equation (10), the most effective way to reduce $\delta a_{m-n}$ is to use a larger proof mass m. However, this will result in an increase in electrostatic voltage to hold the proof mass at its null position, hence bringing more noise from the electrostatic actuator. Using the softer supporting spring with a smaller stiffness $k_{0}$ is another feasible method to reduce $\delta a_{m-n}$. However, it will make $k_{0}$ closer to or even smaller than $k_{e}$, making the spring-mass-damper system unstable in the closed-loop mode. The value *Q* depends on airy viscous damping and materials’ internal damping, thus the capsulation and material of the spring-mass structure should also be considered to get a larger quality factor value. In addition, the spring should be tough enough to support the proof mass, thereby a spring-mass structure with higher stiffness at the cross-axis directions is required to suppress the cross couplings with the sensitive input axis in developing GGI [7,8]. Consequently, all of those issues should be carefully considered when designing a spring-mass structure.

**Figure 2:**
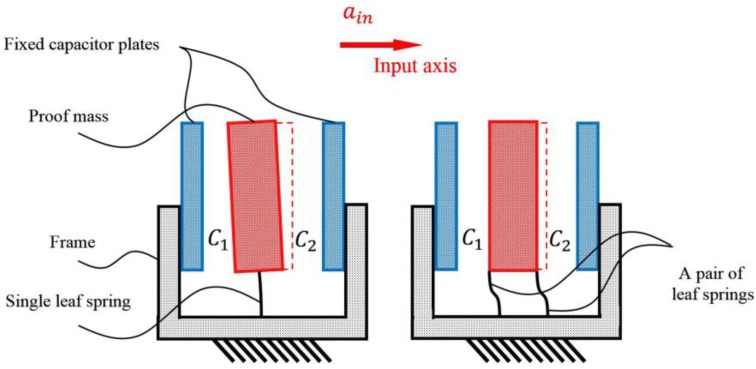
Comparison of the motion of the spring-mass structure with a single leaf spring and a pair of leaf springs, respectively.

In the process of our design and simulation, we found that if the proof mass is inversely supported by two pairs of parallel leaf springs of the same stiffness, the proof mass, centered between two capacitor plates, will move along a true straight line with no swinging motion, thus both capacitor plates are kept parallel. As compared with the single leaf spring structure, as shown in Figure 2, the pairing of two parallel springs not only increase the linearity of the mechanical probe, but also significantly suppress the cross coupling between the sensitive axis and the torsional direction. Furthermore, this structure is strong enough to support a proof mass of up to tens of grams, implying that larger proof masses can be used in this design by adding more pairs of parallel leaf springs with proper stiffness. Because the rotation of the proof mass is almost eliminated, this novel design also ensures the spring-mass structure a very high rejection ratio at the cross-axis directions.

**Figure 3:**
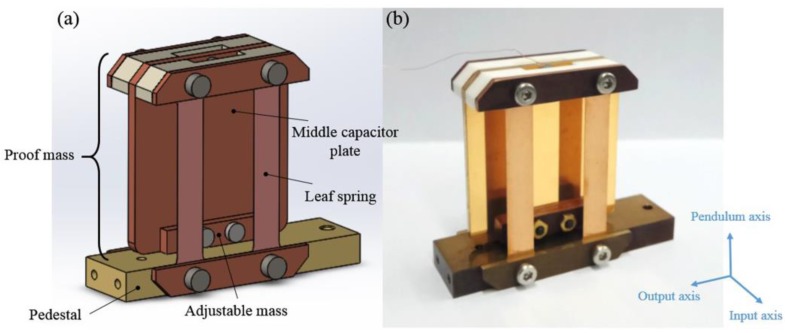
The design and implementation of the proposed spring-mass structure. (**a**) Design drawing of the spring-mass structure. (**b**) The actual spring-mass structure.

The spring-mass structure has been fabricated, as shown in Figure 3. The optimized parameters, with the aim to obtain a low noise and a large cross-axis rejection, are listed in Table 1. The spring-mass structure is fixed to a copper pedestal, and the proof mass with a weight of 27.5 g is inversely supported by two pairs of straight parallel beryllium-bronze leaf springs. An adjustable mass, mounted at the bottom of the proof mass, is used to precisely adjust the centroid of the proof mass. The proof mass is mainly made of the red copper to obtain a heavy mass, and it also acts as a middle capacitor plate coated by a 400 nm gold layer on its surface to prevent oxidization. 

The total stiffness of the leaf springs can be written as

(11) $$k_{0}=\frac{4t^{3}wE}{l^{3}},$$

where w, t, l, and E represent the width, thickness, length, and Young’s modulus of the leaf springs, respectively. 

With the parameters of the proposed spring-mass structure, as shown in Table 1, the static mechanical mode can be analyzed by using the finite element analysis (FEA). The 22 lowest oscillation modes are listed in Table 2 in the order of increasing frequency. The modes marked as I, O, P, $R_{I}$, $R_{O}$, and $R_{P}$ are corresponding to the six degrees of freedom of the spring-mass structure to characterize the motion along the input, output, pendulous axis, and the rotation around the input axis, output, pendulous axis with the frequencies of 7.163, 444.2, 2592, 1458, 1047, 304.5 Hz, respectively. The modes marked as $B_{S1}$, $B_{S2}$, $B_{S3}$ characterize the fundamental, second harmonic, and third harmonic bending modes of the four leaf springs, which are 426, 1192, 2360 Hz, respectively. The mode $R_{S1}$ describes the first order rotation modes of the four leaf springs around the pendulous axis with the frequency of about 1590 Hz. 

According to the FEA results, the proof mass oscillates at a frequency of 7.163 Hz along the input axis at the fundamental vibrational mode I, and the second mode $R_{P}$ is the rotation around the pendulous axis with a frequency of 304.5 Hz. The rejection ratio at the cross-axis directions can be estimated by comparing the stiffness, which can be written as

(12) $$\frac{k_{i}}{k_{I}}=\frac{\omega_{i}{}^{2}/m}{\omega_{I}{}^{2}/m}=\frac{\omega_{i}{}^{2}}{\omega_{I}{}^{2}},$$

where $k_{i}$, $\omega_{i}$ represent the stiffness and angular frequency of the modes at the cross-axis directions, $k_{I}$, $\omega_{I}$ are the stiffness and angular frequency of the fundamental mode I. Therefore, the minimum rejection ratio at the cross-axis directions is $\omega_{R_{P}}{}^{2}/\omega_{I}{}^{2}\approx 1807$, which is just the ratio of the effective spring stiffness of the mode at the cross-axis directions to the fundamental vibrational mode I. It is about four to five times higher when compared to the traditional force-balanced flexure accelerometer. As the proof mass is sandwiched by the two capacitor plates symmetrically, the modes O, P, $R_{I}$ could not influence the capacitive displacement transducer because there is no variation of the differential capacitors. The rotation mode around the output axis $R_{O}$ has a frequency of 1047 Hz, and the rejection ratio is $\omega_{R_{O}}{}^{2}/\omega_{I}{}^{2}\approx 21365$. Finally, the modes $B_{S1}$, $B_{S2}$, $B_{S3}$, $R_{S1}$ are just the vibrations of the leaf springs and will not affect the displacement detection either.

According to Equation (10), the mechanical NEA $\delta a_{m-n}$ of the spring-mass structure is $0.53/\sqrt{Q}\;\mathrm{ng}/\sqrt{\mathrm{Hz}}\;\left(T=300\;K\right)$. If one hopes to obtain a mechanical NEA below 0.3 $\mathrm{ng}/\sqrt{\mathrm{Hz}}$, the quality factor *Q* should be greater than 3, which can be easily achieved by mounting the spring-mass structure into a small vacuum cavity. Furthermore, a quality factor of 30 will further reduce mechanical NEA to below 0.1 $\mathrm{ng}/\sqrt{\mathrm{Hz}}$. 

### 2.3. Noise Analysis of the Sensing and Feedback Circuit

**Figure 4:**
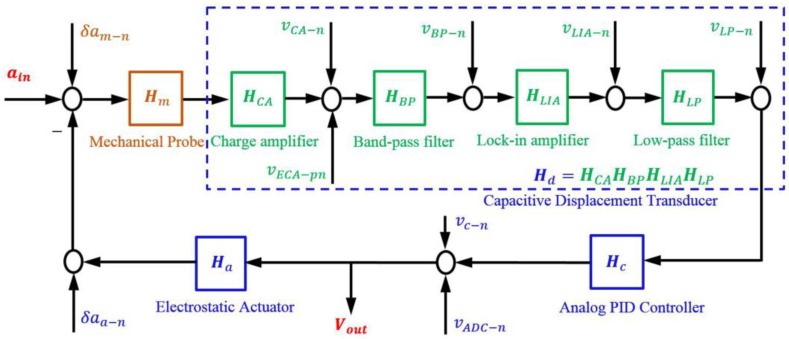
Noise model of the accelerometer. $a_{in}$ denotes the external acceleration, and $V_{out}$ represents the output voltage of accelerometer.

To evaluate the NEA of the circuit $\delta a_{e-n}$, we present a noise model for the accelerometer, as shown in Figure 4. The circuit of the accelerometer contains three parts: the capacitive displacement transducer $H_{d}$, the analog PID controller $H_{c}$, and the electrostatic actuator $H_{a}$, respectively. The capacitive displacement transducer, shown in the dashed line box, is similar to that of Josselin V et al., and Hu M et al. [18,19]. This design consists of a charge amplifier, a band-pass filter, a lock-in amplifier, and a low-pass filter with transfer functions $H_{CA}$, $H_{BP}$, $H_{LIA}$, and $H_{LP}$, respectively. The low-frequency variation of the capacitance of the two differential capacitors, which represents the external acceleration to be measured, is carried by a high-frequency sine wave $v_{p}$ and is input to the charge amplifier by a differential transformer. Through the signal conditioning of band-pass filter, this differential variation of the capacitance is then demodulated by the lock-in amplifier and finally filtered by the low-pass filter. 

According to Figure 4, there are nine types of noise sources that contribute to the total noise of the accelerometer. As the mechanical noise $\delta a_{m-n}$ has been discussed in Section 2.2, here we investigate electronic noise, consisting of the other eight types of noise sources, namely the charge amplifier’s voltage noise $v_{CA-n}$, the sine wave generator’s equivalent voltage noise at the output of charge amplifier $v_{ECA-pn}$, the band-pass filter’s voltage noise $v_{BP-n}$, the lock-in amplifier’s voltage noise $v_{LIA-n}$, the low-pass filter’s voltage noise $v_{LP-n}$, the analog PID controller’s voltage noise $v_{c-n}$, the ADC’s voltage noise $v_{ADC-n},$ and the electrostatic actuator’s NEA noise $\delta a_{a-n}$ which also contains the noise of the regulated DC power supply. Therefore, the power spectral density (PSD) of the electronic noise $\delta a_{e-n}$ could be deduced as

(13) $${\left(\delta a_{e-n}\right)}^{2}\approx {\left(\frac{v_{ECA-pn}}{H_{m}H_{CA}}\right)}^{2}+{\left(\frac{v_{CA-n}}{H_{m}H_{CA}}\right)}^{2}+{\left(\frac{v_{BP-n}}{H_{m}H_{CA}H_{BP}}\right)}^{2}+{\left(\frac{v_{LIA-n}}{H_{m}H_{CA}H_{BP}H_{LIA}}\right)}^{2}+\;{\left(\frac{v_{LP-n}}{H_{m}H_{CA}H_{BP}H_{LIA}H_{LP}}\right)}^{2}+{\left(\frac{H_{a}v_{c-n}}{H_{open}}\right)}^{2}+{\left(\frac{H_{a}v_{\mathrm{ADC}-n}}{H_{open}}\right)}^{2}+{\left(\delta a_{a-n}\right)}^{2}.$$

where $H_{open}=H_{m}H_{CA}H_{BP}H_{LIA}H_{LP}H_{c}H_{a}$.

To get an accurate noise calculation, each part of the noise from the circuit should be carefully considered. The noise of the sine-wave generator is different from the other seven parts because it is related to the imbalances of the capacitance bridge and transformer bridge, and it will be discussed individually in the first place. The noise contributions from the other seven stages are discussed subsequently by using a method similar to that used in [18,19].

The sine-wave generator’s equivalent voltage noise at the output of charge amplifier can be written as

(14) $$v_{ECA-pn}=\frac{2∆C}{C_{f}}v_{p-n},$$

where $v_{p-n}$ is the voltage noise of the sine-wave generator, ∆C is the variation of the capacitance of the differential capacitors, and $C_{f}$ is the feedback capacitor of the charge amplifier. In the closed-loop mode, because of the integral effect of the PID controller, the displacement of the proof mass is approximately equal to zero so that there is almost no change in capacitance. Thus, the noise $v_{EAC-pn}$ can be ignored ideally. However, in fact, there are imbalances of the differential capacitors $C_{1}$, $C_{2}$ and the inductances of the primary windings $L_{1}$, $L_{2}$, which generate a bias capacitance

(15) $$C_{bias}=\frac{C_{1}L_{1}-C_{2}L_{2}}{2L}.$$

This bias capacitance may lead to an increase of the noise $v_{EAC-pn}$ as $∆C=C_{bias}$. According to the first item of Equation (13), the NEA of the noise from the sine-wave generator can be written as

(16) $$\delta a_{p-n}=\frac{v_{ECA-pn}}{H_{m}H_{CA}}=\frac{2C_{bias}}{H_{m}H_{CA}C_{f}}v_{p-n}.$$

With the demand $\delta a_{p-n}<0.3\;\mathrm{ng}/\sqrt{\mathrm{Hz}}$ and the typical parameters of $\left|H_{m}\right|\approx 24,500\;\mathrm{pF}/g$, $H_{CA}C_{f}=10\;V$ and $v_{p-n}=5\;\mathrm{uV}/\sqrt{\mathrm{Hz}}$, the $C_{bias}<7.4\;\mathrm{pF}$ is desired. Besides, the bias capacitance $C_{bias}$ also contributes a bias acceleration $a_{bias}$, which is

(17) $$a_{bias}=\frac{C_{bias}}{H_{m}}.$$

Because $a_{bias}<1\;\mathrm{mg}$ is required in the development of GGI, the equivalent bias capacitance $C_{bias}<24.5\;\mathrm{pF}$ is desired. In this work, the imbalance of the capacitance bridge is compensated by selecting a transformer with suitable parameters to make $C_{bias}$ below 0.5 pF.

The noises of the other seven parts of the circuit are originated from several basic noise sources including:

- the input voltage noise $e_{n}$ and input bias current noise $i_{n}$ of the amplifier,
- the thermal voltage noise $e_{r}$ generated by the resistor,
- the thermal noise $i_{l}$ of the transformer bridge caused by hysteresis loss, eddy current loss, and residual loss of the transformer.

**Figure 5:**
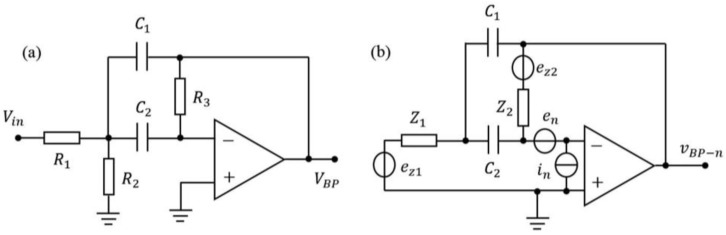
Noise analysis of the band-pass filter. (**a**) Schematic diagram of the band-pass filter. $V_{in}$ and $V_{BP}$ are the input and output voltage of band-pass filter, respectively. (**b**) Noise model of the band-pass filter. The input of band-pass filter is grounded. $Z_{1}=R_{1}//R_{2}$ and $Z_{2}=R_{3}$.

For example, from the noise model of the band-pass filter shown in Figure 5, the basic noise sources are the input voltage noise $e_{n}$ and the input bias current noise $i_{n}$ of the amplifier, and the thermal voltage noises $e_{z1}$, $e_{z2}$ generated by the resistors $Z_{1}=R_{1}//R_{2}$ and $Z_{2}=R_{3}$. If the noise components are uncorrelated, the variances are added as

(18) $$v_{BP-n}=\sqrt{{\left(H_{z1}e_{z1}\right)}^{2}+{\left(H_{z2}e_{z2}\right)}^{2}+{\left(H_{en}e_{n}\right)}^{2}+{\left(H_{in}i_{n}\right)}^{2}},$$

where $H_{z1}$, $H_{z2}$, $H_{en}$, $H_{in}$ are the transfer functions from $e_{z1}$, $e_{z2}$, $e_{n}$, and $i_{n}$ to the output of band-pass filter. The noise caused by the other six parts of the circuit can be discussed in the same way, as introduced by references [18,19], and the basic noise sources of each part are different. To get an accurate result of noise analysis, every basic noise sources ($e_{n}$, $i_{n}$, $e_{r}$, $i_{l}$) of the seven parts are evaluated and measured to meet the requirements in the process of making and debugging the sensing and feedback circuit.

### 2.4. Noise Analysis Result

**Figure 6:**
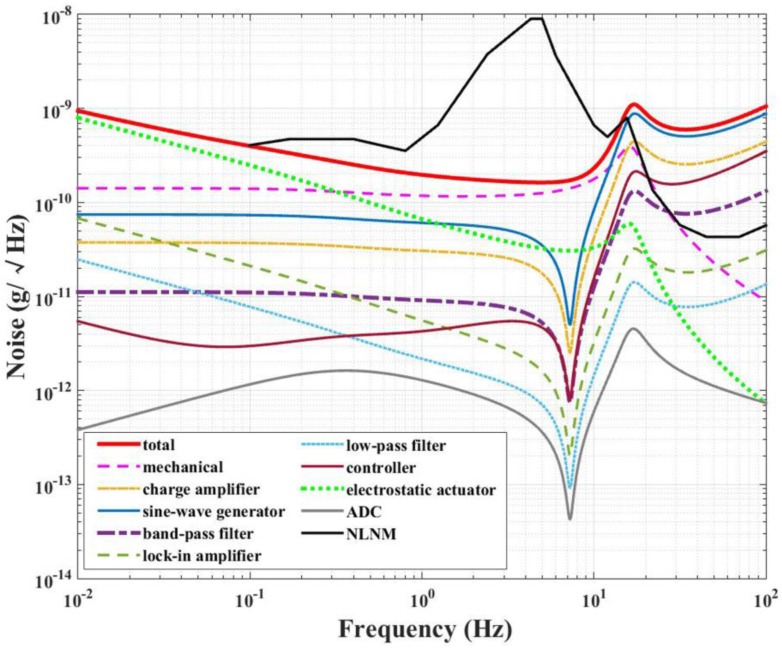
Noise analysis result of the electrostatic accelerometer. The black solid line shows the new low-noise model (NLNM) presented by Peterson [25], and the red solid line shows the calculated total noise of the proposed accelerometer, which is lower than NLNM over the frequency range of 0.1 to 10 Hz.

Based on the analysis in the previous section, the contributions to the mechanical noise $\delta a_{m-n}$ and electronic noise $\delta a_{e-n}$ are both taken into consideration to meet the requirement of GGI. According to Equation (13), the electronic noise of the instrument can be optimized by adjusting the gain of the transfer functions of the sensing and feedback circuit. Finally, the optimized result is shown in Figure 6. It can be seen that the NEA of the electrostatic actuator $\delta a_{a-n}$ dominates the total noise below 0.3 Hz. From 0.3 to 10 Hz, the mechanical noise $\delta a_{m-n}$ becomes more significant. The total noise of the instrument is calculated to be between $0.2\;\mathrm{ng}/\sqrt{\mathrm{Hz}}$ and $0.3\;\mathrm{ng}/\sqrt{\mathrm{Hz}}$ over the frequency range from 0.2 to 2 Hz, and it is below the new low-noise model (NLNM) over the frequency range of 0.1 to 10 Hz [25].

## 3. Prototype and Preliminary Test Results

**Figure 7:**
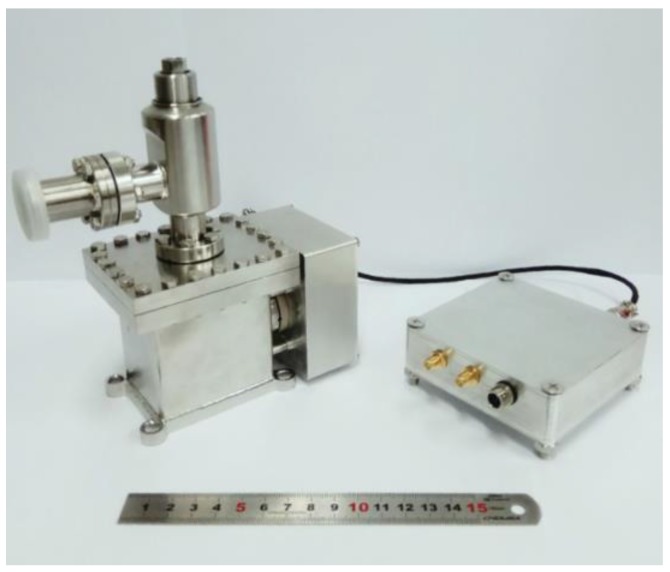
The prototype of the accelerometer. The spring-mass structure is encapsulated in a small vacuum cavity (**left**) and the circuit is installed in a shielding box (**right**).

Based on the design, the prototype of the accelerometer was fabricated, as shown in Figure 7. All of the characterization of the accelerometer was achieved in the cave laboratory of HUST, in which the variation of temperature is lower than 0.03 K/day and the vibration noise floor is extremely low [26].

### 3.1. Measurement of Quality Factor and Natural Frequency

**Figure 8:**
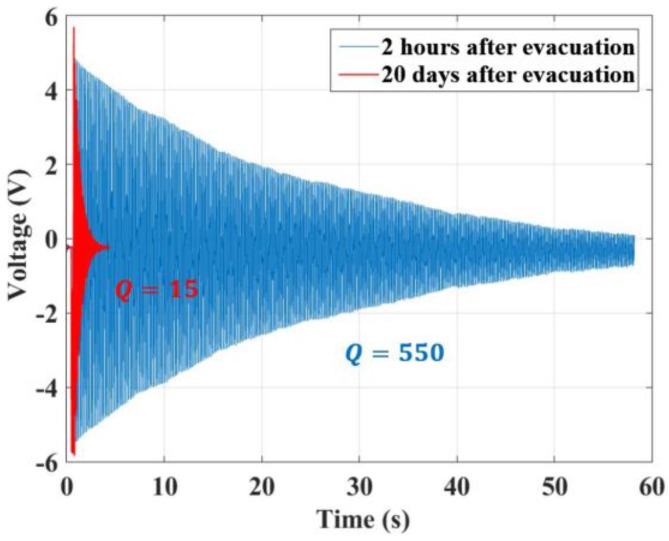
Pulse response curve of the accelerometer in the time domain. Pulses were supplied by the electrostatic actuator. The blue and red curves show the pulse responses at 2 h (*Q* = 550) and 20 days (*Q* = 15) after evacuation, respectively.

We firstly measured the quality factor *Q* and the natural frequency $f_{0}$. In the accelerometer design, the spring-mass structure was assembled in a small vacuum cavity where the air pressure can be maintained at a low level without the ion pump and getter. The pulse response curve of the spring-mass structure, as shown in Figure 8, indicates that the quality factor *Q* was about 550 at 2 h after evacuation. After 20 days, it dropped down to 15. The quality factor *Q* has been maintained at 15 for six months until the time when this manuscript is being prepared. The natural frequency $f_{0}$ was kept at 7.35 Hz. Thus, the actual $\delta a_{m-n}$ can be easily given as $\delta a_{m-n}=0.14\;\mathrm{ng}/\sqrt{\mathrm{Hz}}$ (*Q* = 15), which agrees with the predicted result within 17%.

### 3.2. Calibration of Accelerometer’s Scale Factor

**Figure 9:**
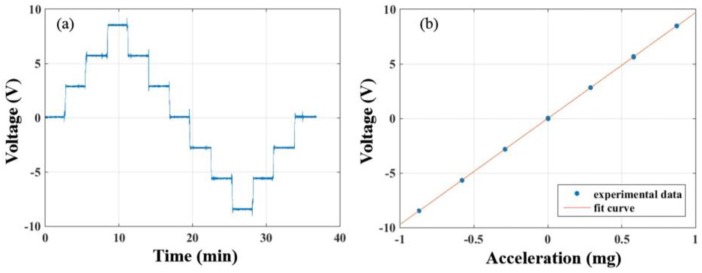
The results of calibration of accelerometer’s scale factor (**a**) Calibration curve with the step about 0.29 mg; (**b**) Result of linear fit with the scale factor K=(9725±11) V/g and the nonlinearity of about 0.1%.

Next, we calibrated the accelerometer’s scale factor by a tilt method. In this experiment, a biaxial dividing head with a precision of one arcsecond was applied to change the accelerometer’s orientation in order to detect the variation of input acceleration that is projected by the local gravity vector. The dividing head is rotated by $1^{\prime }$ each time so that the variation of input acceleration is about 0.29 mg per step. The rotating angle is $\pm 1^{\prime }$, $\pm 2^{\prime }$, $\pm 3^{\prime }$, and the calibration curve is shown in Figure 9a. By analyzing the data with a linear fitting as shown in Figure 9b, the scale factor of the accelerometer can be calculated as

K=(9725±11) V/g

with a nonlinearity of about 0.1%. The measuring range of the present accelerometer is set to be ±1 mg, corresponding to an output voltage of about ±9.725 V.

### 3.3. Estimation of Self-Noise of the Accelerometer

To measure the self-noise of a high-resolution accelerometer, experiments are usually conducted in seismically quiet areas like desert, cave, vibration isolation platform etc. However, as discussed in Figure 6, the predicted noise of accelerometer is lower than NLNM, making it very difficult to measure the actual noise in the environment with a large seismic noise. To overcome this challenge, we introduce a method that is based on coherence analysis. If the test accelerometer X and the reference accelerometer Y are of the same type, then the self-noise of these two accelerometers could be treated as identical. Thus, when the two accelerometers are aligned together in the same direction, both of them are detecting a coherent seismic noise. Therefore, the coherence analysis can be used to eliminate the coherent seismic noise, and then the self-noises of the two accelerometers can be written as [27,28]

(19) $$\left|NEA_{n}\left(\omega \right)\right|=\left|NEA_{X}\left(\omega \right)\right|\sqrt{\left[1-\sqrt{\gamma_{XY}^{2}\left(\omega \right)}\right]},$$

where $NEA_{X}\left(\omega \right)$ is the NEA of the accelerometer X, which contains the self-noise of the instrument and the seismic noise. $\gamma_{XY}^{2}\left(\omega \right)$ is the coherence between the measured outputs of the accelerometers X and Y.

**Figure 10:**
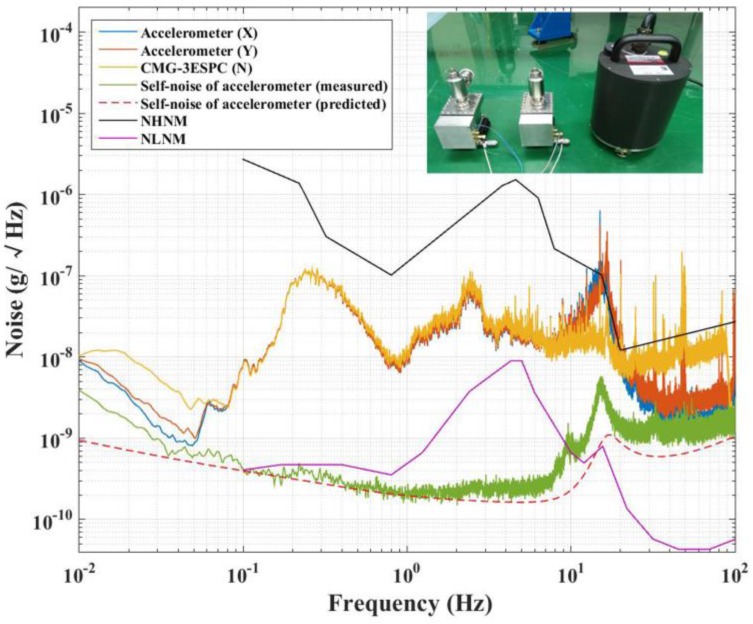
Instrument’s noise test result of the electrostatic accelerometer. The black and pink solid lines are the noise equivalent acceleration (NEA) of the new high-noise model (NHNM) and NLNM, as proposed by Peterson [25], respectively. Besides, the blue solid line, orange solid line, and yellow solid line are separately the NEA of the signals acquired by test accelerometer (X), reference accelerometer (Y), and CMG-3ESPC, which all contain the NEA of the seismic noise and the instruments. The green solid line shows the NEA of the accelerometer, which is based on coherence analysis. The red dash line shows the NEA of the accelerometer predicted in Section 2.4.

However, in practice, there is always a misalignment between the two accelerometers. This misalignment brings an incoherent seismic noise that cannot be reduced by the coherence analysis. To solve this problem, a commercial triaxial seismometer is used to find the orientations of the two accelerometers [29]. Since $\vec{a_{X}}$, $\vec{a_{Y}}$ represent the seismic noise tested by the two accelerometers, an acceleration vector ${\vec{a_{YX}}}^{\prime }$, which can be found by iterative algorithms with the criterion of the coherence of $\vec{a_{X}}$ and $\vec{a_{Y}}+{\vec{a_{YX}}}^{\prime }$ [27,28,29], is used to align the orientation of reference accelerometer Y. Finally, the coherent seismic noise can be almost eliminated by the coherence analysis. In this test, the commercial triaxial seismometer, CMG-3ESPC, is applied to compensate for the misalignment. The experimental results are shown in Figure 10. Finally, the measured self-noise is between 0.2 $\mathrm{ng}/\sqrt{\mathrm{Hz}}$ and 0.3 $\mathrm{ng}/\sqrt{\mathrm{Hz}}$ over the frequency range of 0.2 to 2 Hz, which agrees with our prediction.

## 4. Conclusions

An electrostatic accelerometer is under development with a demonstrated resolution of 0.2 $\mathrm{ng}/\sqrt{\mathrm{Hz}}$ to 0.3 $\mathrm{ng}/\sqrt{\mathrm{Hz}}$ over the frequency ranging from 0.2 to 2 Hz. Experimental results show that a scale factor of (9725±11) V/g and a measurement range of ±1 mg with the maximal output voltage of about ±10 V, can be achieved. The minimum rejection ratio between the fundamental vibrational mode and the other modes at the cross-axis directions $\omega_{i}{}^{2}/\omega_{I}{}^{2}$ is 1807, which is four times higher than the conventional force-rebalanced flexure accelerometers. In summary, the performance of the proposed electrostatic accelerometer meets the demands in developing the rotating accelerometer gravity gradiometer.

When compared to the previous STAR, SuperSTAR and GOCE electrostatic-space-accelerometers [13], our accelerometer has a wider measurement range, a smaller volume, and a higher cut-off frequency, and is able to work in near-earth gravity environment. The commercial seismometers based on the principle of speedometer, such as Nanometrics’ Trillium compact HP, Guralp’s CMG-3T, and Streckeisen’s STS-2, indeed have a lower noise floor [14,15,16,17]. However, their bulky size and extremely high costs are major disadvantages as compared to our accelerometer. Furthermore, the self-noise of our accelerometer is almost three orders lower than typical inertial navigation-grade accelerometers, such as Honeywell’s QA-3000 [30]. Although the long-time stability of our accelerometer is not as good as QA-3000, it has met the requirement of being used in GGI. Indeed, the noise floor of our accelerometer is still higher than the improved Bell model VII, which has been applied to the Bell’s GGI. Nevertheless, it meets the noise requirement of $0.3\;\mathrm{ng}/\sqrt{\mathrm{Hz}}$ over the frequency range of 0.2 to 2 Hz for developing our GGI at the present stage. Efforts can also be made to further decrease the noise floor of the proposed accelerometer.

## Figures and Tables

**Table 1:** A typical set of parameters of the proposed spring-mass structure.

Parameter	Value
Proof mass m (g)	27.5
Leaf springs’ w, t, l (mm)	5, 0.08, 28
Leaf springs’ Young’s modulus E ($N/m^{2}$)	$1.25\times 10^{11}$
Total stiffness $k_{0}$ (N/m)	58.3
Natural frequency $f_{0}$ (Hz)	7.33

**Table 2:** The 22 lowest oscillation modes of the proposed spring-mass structure.

**NO.**	**1**	**2**	**3, 4, 5, 6**	**7**		**8**
Frequency (Hz)	7.163	304.5	425.4, 425.7 426.0, 426.7	444.2		1047
Description of mode (i)	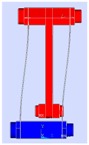 I	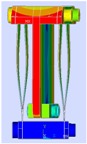 $R_{P}$	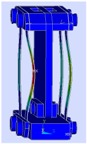 $B_{S1}$	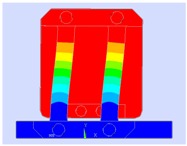 O		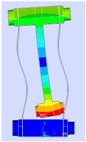 $R_{O}$
Rejection ratio $\omega_{i}{}^{2}/\omega_{I}{}^{2}$	1	1807	3527, 3540 3540, 3556	3846		21365
**NO.**	**9, 10, 11, 12**	**13**		**14, 15, 16, 17**	**18, 19, 20, 21**	**22**
Frequency (Hz)	1191, 1192 1192, 1196	1458		1588, 1589 1590, 1591	2359, 2360 2363, 2368	2592
Description of mode (i)	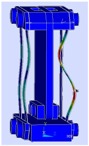 $B_{S2}$	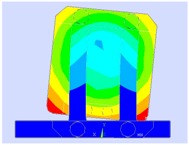 $R_{I}$		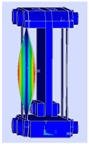 $R_{S1}$	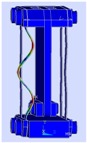 $B_{S3}$	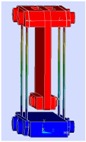 P
Rejection ratio $\omega_{i}{}^{2}/\omega_{I}{}^{2}$	27,646, 27,692 27,692, 27,879	41,431		49,148, 49,210 49,272, 49,334	108,459, 108,551 108,827, 109,288	130,942
